# Oral Metronomic Chemotherapy in Advanced and Metastatic Oral Squamous Cell Carcinoma: A Need of the Hour

**DOI:** 10.1007/s12663-023-01963-y

**Published:** 2023-07-04

**Authors:** Naveena A. N. Kumar, Punit Singh Dikhit, Anmi Jose, Vedant Mehta, Ananth Pai, Adarsh Kudva, Mahadev Rao

**Affiliations:** 1 Department of Surgical Oncology, Manipal Comprehensive Cancer Care Centre, Kasturba Medical College, Manipal Academy of Higher Education (MAHE), Manipal, Karnataka 576104 India; 2 Department of Pharmacy Practice, Manipal College of Pharmaceutical Sciences, Manipal Academy of Higher Education (MAHE), Manipal, 576104 India; 3 Department of Medical Oncology, Manipal Comprehensive Cancer Care Centre, Kasturba Medical College, Manipal Academy of Higher Education (MAHE), Manipal, Karnataka 576104 India; 4 Department of Oral and Maxillofacial Surgery, Manipal College of Dental Sciences, Manipal Academy of Higher Education (MAHE), Manipal, Karnataka 576104 India; 5 Department of Pharmacy Practice, Manipal College of Pharmaceutical Sciences, Manipal Academy of Higher Education (MAHE), Manipal, Karnataka 576104 India

**Keywords:** Oral metronomic chemotherapy, Oral cancer, Oral squamous cell carcinoma, OMCT, Systemic therapy

## Abstract

**Aim:**

The present review article aims to compile the best available evidence-based data on oral metronomic chemotherapy (OMCT) including its mechanism of action, its utility, and future directions.

**Methods:**

A systematic search was carried out in PubMed database for available English literature from last 10 years between 2011 and 2021. Keyword combinations used were ‘Oral Metronomic chemotherapy for oral cancer, mechanism of action of OMCT, Oral metronomic chemotherapy in India, OMCT in recurrent and palliative treatment of oral cancers.’

**Results:**

Multitudes of studies have been published recently stating the role of OMCT in head and neck squamous cell carcinoma (HNSCC), but the studies with the category of level of evidence required to advocate OMCT as a recognized therapy are still scarce. On careful stratification of these studies, we found that OMCT has a lot to offer in palliative settings, recurrent, and metastatic HNSCC. There is some limited evidence of its role in adjuvant therapy as maintenance and in neoadjuvant setting.

**Conclusion:**

With current evidence, there is a definite role of OMCT in treatment of oral SCC. OMCT can be an alternative in patients who are not tolerable or affordable for standard palliative chemotherapy and also an option for patient who are waiting for surgery. However, results of ongoing and future studies on exact mechanism, indications, and implications of this drug regimen would help in integration OMCT in current standard of therapy.

**Graphical abstract:**

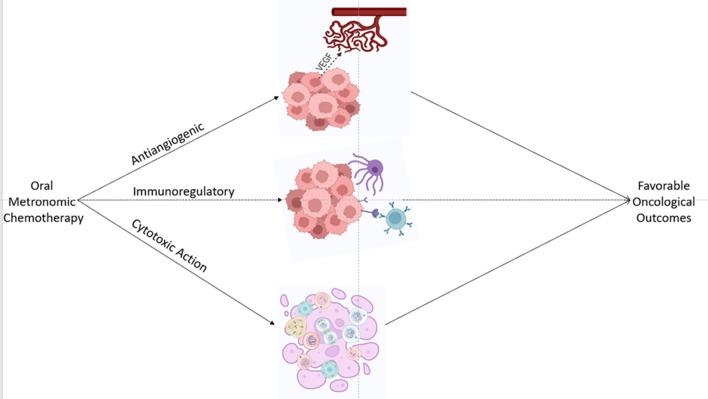

## Introduction

Head and neck squamous cell carcinoma (HNSCC) is one of the common malignancies in Asian subcontinents [1]. According to GLOBOCAN 2020, HNSCC is the third most common cancer in India with its incidence amongst males being second most common [2, 3]. Around 60–80% of these cases present in an advanced stage [3, this problem is further complicated by non-availability of tertiary cancer centres in every region of India and financial burdens which increases the time between the diagnosis and definitive treatment [2]. These issues warrant the use of therapies that would prevent the relentless progression of the malignancy, promote its regression, and make sure that the disease remains operable while awaiting definitive treatment which in most cases is surgery [4]. Such therapies would also be required to be easily deliverable, nontoxic, and cost-effective. The conventional chemotherapy cannot step into the shoes for this sort of arrangements, firstly because conventional chemotherapy can cause significant toxicity. Secondly, these therapies also require the rest periods between cycles of therapy, which can lead to tumor cell repopulation.

Oral metronomic chemotherapy (OMCT) is an alternative that fulfils above criteria including economical cost, can be given in low doses with regular administration at shorter interval without interruption. The term “metronomic” basically means the musical device “metronome” that is known to produce regular short ticks representing regular aural pulse which is similar to the idea of metronomic chemotherapy that is regular administration of the chemotherapeutic drugs resulting in constant low blood level of the drug. The usual recommended drugs for OMCT includes daily dose of celecoxib 200 mg twice a day and oral low dose methotrexate, i.e., 15 mg/m^2^ weekly. The therapy is usually continued till surgery in advanced cancer or till progression in metastatic disease, intolerable side effects or life-threatening complication [5].

The present review article aims to compile the best available evidence-based data on OMCT including its mechanism of action, its utility, and future directions.

## Materials and Methods

**Fig. 1:**
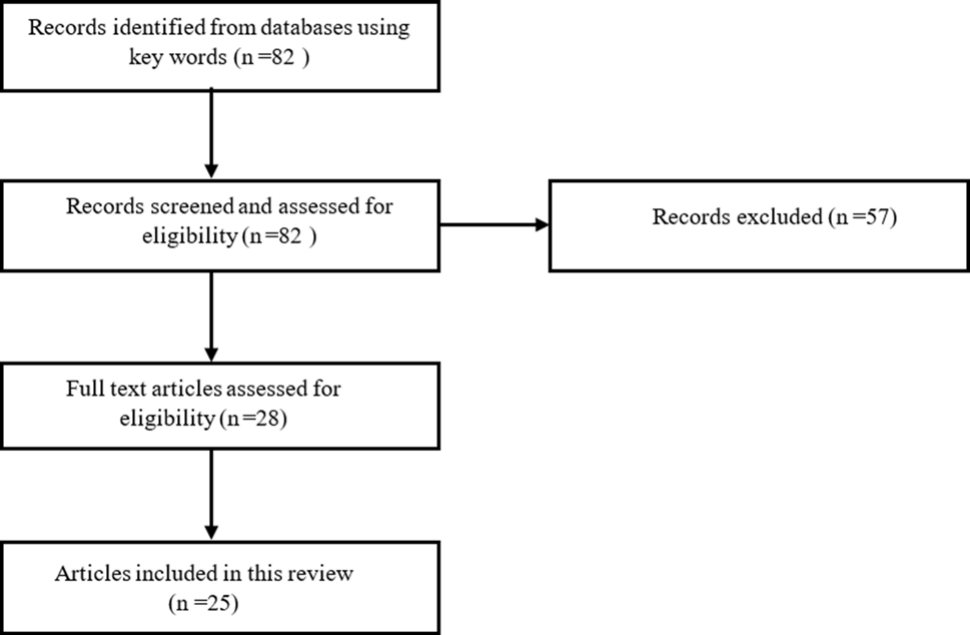
Flow chart of the article selection

A systematic search was carried out in PubMed database for available English literature from last 10 years between 2011 and 2021. Keyword combinations used were “Oral Metronomic chemotherapy for oral cancer, mechanism of action of OMCT”, Oral metronomic chemotherapy in India, OMCT in recurrent and palliative oral cancers. The flow chart of article selection for review is shown in Fig. 1.

## Mechanism of Action of OMCT

Over the past few years, there has been an exceptional growth in the literature and knowledge related to principals of molecular pathways and response of tumor cells to conventional chemotherapy. Few things which became clear includes the fact that the cells of solid neoplasms usually comprise the heterogeneous group with divergent cell kinetics and angiogenic potential. Secondly, tumor cells can attain resistance to chemotherapy because of their intrinsic genomic instability. Lastly, metastases are one of the common reasons for relapse of tumor and tumor-related death, so conventional chemotherapy is usually less effective against metastases compared to the primary [6].

**Fig. 2:**
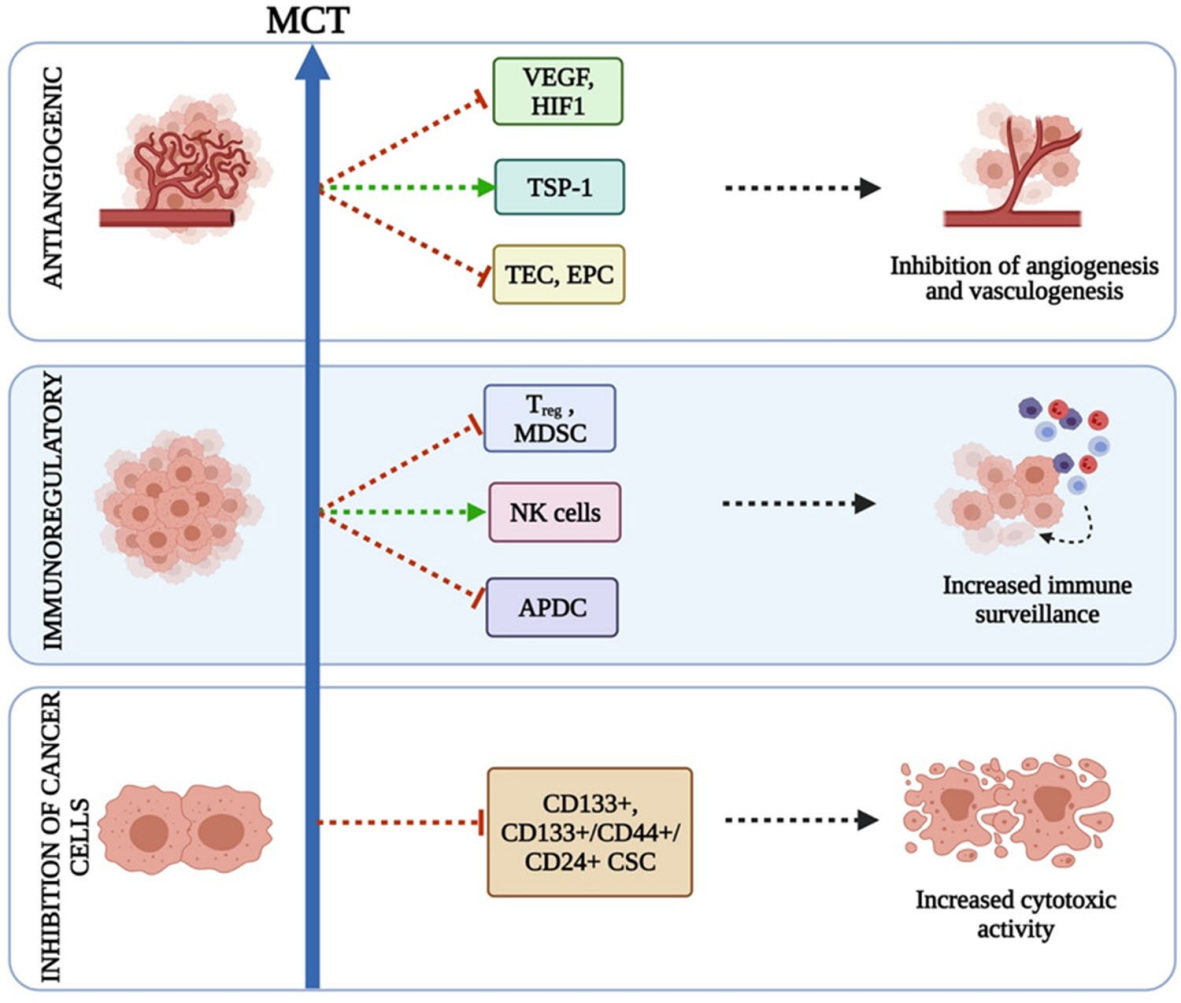
Proposed mechanism of action of OMCT. VEGF: vascular endothelial growth factor; TSP-1: thrombospondin 1; TEC: tumor endothelial cells; EPC: endothelial progenitor cells; T regs: regulatory T cells; MDSC: myeloid-derived suppressor cell; NK cells: natural killer cells; APDC: antigen presenting dendritic cell; CSC: cancer stem cells

OMCT works by antiangiogenesis, modulating immune response and inducing tumor cells inactivity. The mechanism of OMCT is depicted in Fig. 2. The drugs targeting angiogenic pathways damage the tumor cells by the induction of hypoxia and nutrient starvation. The similar mode of action is shared by another class of therapy, i.e., anti-VEGF monoclonal antibodies. However, it possesses many different aspects. The antiangiogenic drug directly targets the action of vascular endothelial growth factor (VEGF), while the OMCT disables the cells involved in the angiogenic mechanisms, implicating that the tumoral endothelial cells (TEC) could be a better target to overcome the drug resistance. Apart from its prominent effects on endothelial cells, MCT also promotes induction of antiangiogenic protein Thrombospondin-1, which in turn inhibits angiogenic HIF-1α, and decreases circulating VEGF levels [7].

Immunity has a vital role to play in cancer, with escape from immunosurveillance being one of the most important factors for the growth of cancer. Conversely, an active functioning immune system is indispensable for the optimal actions of chemotherapy agents. Both adoptive and innate immunity plays an important role in the cancer cells growth arrest. Cancer cells are known to escapes immune surveillance. Chemotherapeutic agents are known to induce dendritic cell maturation, although the exact mechanism involved in case of OMCT is yet to be elucidated [6, 7].

But the current most accepted proposed mechanism of action involves regulatory T cells (Tregs). Tregs are known CD4 + , CD25 + lymphocytes, which contains glucocorticoid-induced tumor necrosis factor (TNF) receptor, and cytotoxic T lymphocyte associated antigen 4 that inhibit antitumor immune response by inhibiting both tumor-specific and nonspecific natural killer (NK) cells. Tregs have been shown to be increased in multiple cancers. Ghiringhelli et al. demonstrated potent activity of low dose of cyclophosphamide (CTX) against circulating Tregs and their immunosuppression in various human cancers. They also stated that lower doses of CTX can led to the restoration of the immune-specific cytotoxic T cells activity and NK cell’s function. The regular doses of chemotherapeutic agents inhibit the activities of all immune cells thus it lacks the Tregs-specific actions. OMCT in low doses exhibits Tregs-specific activity and is an active area of research in terms of cancer immunity and drugs interactions [8].

## OMCT Drugs

The two key drugs suggested in multiple literature for OMCT are celecoxib and methotrexate. Mechanism of action for celecoxib basically hinges around cyclooxygenase-2 (COX-2) inhibitor. Majority of head and neck malignant tumors presents with overexpression of COX-2. Evidence also supports the fact that COX-2 overexpression is responsible for tumorigenesis [9]. Also, COX-2 overexpression was responsible for lymph node metastasis in head and neck cancer patients. Studies suggested that overexpression of COX-2 is associated with lymph node metastasis, which in turn results in low survival rates. The initial events in cancer progression are alterations in cell adhesion, including cell–cell junctions as well as cell-extracellular matrix (ECM) interactions. The orderly structured stratification of epithelial tissues is mostly maintained by E-cadherin and N-cadherin. E-cadherin transcription is usually blocked by the transcription factors Slug, Snail and Twist, while N-cadherin is significantly raised by Snail and Twist. Exogenous prostaglandin treatment led to upregulation of Snail and ZEB1 and downregulation of E-cadherin. These findings indicate that abnormal COX-2/PGE2 overexpression is involved in cancer development [10, 11].

Methotrexate is the second key drug involved in OMCT. It is the 4-amino 10-methyl analog of folic acid. It binds and inhibits dihydrofolate reductase, which is a critical enzyme in maintenance of intracellular folates in reduced form. This reduced state is important for synthesis of nucleic acid, the deficiency of which results in DNA strands breaks. It also acts by direct inhibition of folate dependent enzymes and by incorporating aberrant nucleotides into DNA, resulting in inhibition of DNA synthesis.

## Discussion

Multitudes of studies have been published recently stating the role of OMCT in HNSCC, but the studies with the category of level of evidence required to advocate OMCT as a recognized therapy are still scarce. On careful stratification of these studies, we found that OMCT has a lot to offer in palliative settings, recurrent, and metastatic HNSCC. There is some limited evidence of its role in adjuvant therapy as maintenance and in neoadjuvant setting. The summary of the studies in palliative and curative setting is depicted in Tables 1 and 2, respectively.

**Table 1:** OMCT in palliative settings

Study	Disease setting/study population	Type of study	Number of patients	Treatment regimen	Outcomes	Results
Patil VM et al. 2015[5]	Metastatic, relapsed or inoperable HNSCC	Phase II	110	Celecoxib 200 mg twice daily + methotrexate 15 mg/m^2^ weekly (*n* = 57) versus cisplatinum 75 mg/m^2^, 3 weekly (*n* = 53)	OS, PFS	PFS 101 versus 66 days (*p* = 0.014); OS 249 versus 152 days (*p* = 0.02)
Geetha et al. 2016[9]	Recurrent/metastatic	Single arm prospective observational study	15	Methotrexate 15 mg/m^2^/week, oral celecoxib 200 mg twice daily and erlotinib 150 mg once daily	PFS	PFS:148 days
K.Kalaichelvi,2016[9]	Advanced/recurrent head and neck squamous cell carcinoma (HNSCC)	Retrospective	30	Weekly oral methotrexate 5 mg twice daily for 2 days/week and oral celecoxib 200 mg twice daily	CBR, QOL	Pain control: 80%, QOL improved
Patil VM et al. 2020[14]	Relapsed/recurrent or newly diagnosed	Phase III	422	Weekly methotrexate 15 mg/m^2^ + celecoxib 200 mg twice daily (*n* = 213) versus 75 mg/m2 IV cisplatin once every 3 weeks (*n* = 209)	OS	OS 7.5 versus 6.1 months (*p* = 0.026) OMCT was noninferior to IV cisplatinum
Patil VM et al. 2015[15]	Oral squamous cell carcinoma patients with early failure and/or platinum-resistant disease	Retrospective	100	Weekly methotrexate 15 mg/m^2^ + celecoxib 200 mg twice daily	OS, 6 months survival	OS: 110 days, 6 months survival: 26.4%. OMCT failed to meet its prespecified efficacy limit
Shilpa Kandipalli et al. 2018[17]	Recurrent, residual and metastatic head & neck cancers	Prospective observational Study	47	Methotrexate 2.5 mg twice weekly Capecitabine: 500 mg twice daily (*n* = 47) versus baseline	Quality of life	Improved QOL
Patil VM et al. 2017[21]	Unselected cohort of head and neck cancer	Retrospective	340	Methotrexate 15 mg/m^2^ twice weekly + celecoxib 200 mg twice daily	OS	OS: 155 days, Promising results in selected group of patients
Patil VM et al. 2012[22]	Metastatic, recurrent and locally advanced oral cavity cancers which were not amenable to local treatments	Retrospective	18	Weekly methotrexate 15 mg/m^2^ + celecoxib 200 mg twice daily	Toxicity profile and efficacy	Toxicity: minimal, CBR: 44.48%, Symptom control: 66.7%, PFS: 5 months, OS:3.05 months
Kumar KSS et al. 2019[23]	advanced/recurrent HNSCC	Phase II study	50	Methotrexate 15 mg twice daily for 2 days/week + celecoxib 200 mg twice daily	Overall response rate	DCR: 76% (2 months) and CBR:64% (6 months)
V Noronha, VM Patil, 2017[24]	Metastatic/recurrent head and neck squamous cell cancer	Match pair analysis	120	Weekly Paclitaxel 80 mg/m^2^ + cetuximab (60) versus MCT (60)	OS	OS: 191 days in MCT versus 256 days in cetuximab cohort

*N* number, *OS* overall survival, *PFS* progression free survival, *DFS* disease free survival, *DCR* disease control rate, *CBR* clinical benefit rate

**Table 2:** OMCT in advanced oral cancer(operable)–Adjuvant and neoadjuvant

Study	Disease setting/study population	Type of study	Number of patients	Treatment regimen	Outcomes	Results
Pai PS et al. 2013[4]	Advanced operable oral cancers,	Retrospective matched‑pair analysis	OMCT group (*n* = 32) versus control group (*n* = 32)	Oral methotrexate 15 mg/m^2^ once a week (*n* = 32) and oral celecoxib 200 mg twice daily(*n* = 32)	DFS	86.5% versus 71.6%
Pandey A et al. 2016[10]	Resectable oral cavity carcinoma	Retrospective	225	Methotrexate (15 mg/m^2^) once a week and celecoxib 200 mg twice a day daily (*n* = 130) versus versus no MC (*N* = 95)	DFS, OS	DFS (14 versus 8 months, *p* = 0.22) and OS (26 versus 14 months, *p* = 0.04)
Ming-yu et al. 2018[25]	Stage III–IV oral squamous cell carcinoma, and received curative surgery and postoperative chemoradiotherapy	Retrospective	356	Group 1: tegafur-uracil 100–200 mg/day (*n* = 23), Group 2: tegafur-uracil 300–400 mg/day (*n* = 23) vs control (*n* = 242)	DSS, DFS, and OS	OS: 65% versus 48% (*p* = 0.0008) DFS: 57% versus 41% (*p* = 0.0034) DSS: 74% versus 61% (*p* = 0.0029)

*n* number, *OS* overall survival, *PFS* progression free survival, *DFS* disease free survival, *DSS* Disease-specific survival

### OMCT in Palliative Setting

Palliative chemotherapy for head and neck cancers with metastatic, recurrent, or inoperable setting has evolved over time. It started with a small pilot study indicating a survival benefit of chemotherapy over patients treated with only supportive measures. Over next 3–4 decades, we have significantly improved survival rates by adding targeted therapy to conventional chemotherapy. The benefit of targeted therapy, that is cetuximab was seen in multiple trials and studies with selected cohort of patients who were non-responders to platinum-based chemotherapy. Since then, many studies indicated that cetuximab as a single agent seems to provide an appropriate option in these setting. These findings were further strengthened by EXTREME study with median overall survival (OS) of 11 months in patients receiving cetuximab, cisplatin, and 5-fluorouracil [12]**.**

Palliative chemotherapy involves patients who presents with metastatic disease, who fails within 6 months of CT-RT or within 1 month of RT or patients presenting with platinum refractory disease. EXTREME trial and KEYNOTE-048 trials [13] have already set the landmarks and standards of care with targeted therapy for such patients, but access of such drug regimen to general population of Indian subcontinent is still a matter of debate keeping in view the logistic and financial issues. For the same reasons requirement for an alternative therapy, if not ideal are the need of hour where OMCT steps in for these subsets of patients**.**

A prospective randomized phase II study was done by VM Patil et al. in patients requiring palliative chemotherapy which compared oral MCT [daily celecoxib (200 mg twice daily) and weekly methotrexate (15 mg/m2)] and intravenous single agent cisplatin (IP) (75 mg/m2) given 3 weekly. A total of 110 patients were recruited out of which 57 were randomized to the OMCT arm and 53 to the IP arm. They reported that patients in the MCT arm had significantly longer progression-free survival as compared to the IP arm (*p* = 0.014). The overall survival (OS) was also noted to be increased significantly in the OMCT arm when compared to the IP arm. They concluded by stating that OMCT has significantly better outcomes in terms of progression-free survival (PFS) and OS as compared to single agent platinum in the palliative setting [5]**.**

Patil et al. conducted an open-label, parallel group, non-inferiority, randomized, phase 3 trial on patients with recurrent, metastatic, inoperable head and neck carcinoma. A total of 422 patients were included in the study (213 in OMCT group and 209 in cisplatin group). Primary endpoint was overall survival measured from the date of randomization to the date of death or censored to the date of last follow-up. Secondary endpoints included progression-free survival, the number of patients who responded to the therapy, toxicity, and quality of life. At a median follow-up of 15·73 months, median overall survival was 7·5 months for the oral metronomic chemotherapy group compared with 6·1 months in the intravenous cisplatin group. Grade 3 or higher adverse side effects were noted in 19% of 196 patients in the oral metronomic chemotherapy group versus 30% of 202 patients in the intravenous cisplatin group. They concluded that OMCT can be a good alternative standard of care if current National Comprehensive Cancer Network (NCCN)-approved options for palliative therapy are not feasible [14]**.**

Another study was published by Kalaichelvi et al. to evaluate the efficacy and toxicity profile of metronomic chemotherapy in patients with advanced or recurrent HNSCC. Efficacy was noted in terms of clinical benefit rate, pain control, changes in quality of life and median time to progression of disease. Their study suggested that 60% patients had stable disease, 10% had partial response, and 30% had progressive disease. They finally concluded that OMCTs with advanced/recurrent HNSCC was effective and well tolerated while providing good pain control and improves quality of life with least toxicity profile [9].

Based on above proposed mechanism of actions and hypothesis multiple pilot studies and trials have been performed. The findings of these trials pointed toward multiple variables affecting the outcome of OMCT. One of the biggest factors being the time of failure, with better OS in patients with time to failure of > 6 months as compared to failure within 6 months. This subgroup of patients who fail within 6 months are basically patients with platinum refractory disease, who usually performs worse [15]. This data have been consistently duplicated in multiple studies including EXTREME trial where PFS was better in platinum-based chemotherapy arm, the possible reason being the fact that the study excluded the patients with platinum refractory disease that is patients who failed within 6 months [12].This was in contrast to the prospective study by Patil et al. where PFS was only 66 days in chemotherapy-based arm because their study included the patients with chemotherapy free interval of less than 6 months. As compared to chemotherapy arm, OMCT arm performed better in terms of median OS, PFS and adverse outcomes and was noted to be non-inferior to chemotherapy arm [5]. The possible explanation for these better results in platinum refractory cases lies with OMCT’s antiangiogenesis effects and actions on endothelial cells of the vasculature supplying the tumor. Since the endothelial cells of vasculature are basically genotypically stable cells hence it should not be affected by somatic mutations in the tumor which are responsible for platinum-resistance [6, 7, 15].

The other important factor that has been echoed in multiple studies is the role of sites and subsites in OS and PFS. It has been reiterated that advanced or recurrent oral cavity primaries perform worst in terms of OS and PFS as compared to pharyngeal and laryngeal primaries. The general notion is that salvage surgery always provides best outcome as compared to chemotherapy in oral cavity primaries, but only about 16% of such patients present with a stage that is amenable to surgery. Study by Vermorken et al. which stated that patients on cetuximab performed better in terms of OS in palliative settings had a mixed subsets of patients with oral cavity primaries constituting only about 21% in study arm and 19% in control arm [16], while majority of trials and studies included in our review which represents Indian subset of population is majorly comprised oral cavity primaries. So, when compared to other studies, OMCT drug regimen performance is quite satisfactory as compared to established regimen. This difference in response rate between oral cavity and other head and neck primaries (laryngeal and pharyngeal) also needs further evaluation in terms of HPV association and other factors.

Lastly, one important factor that tips the balance toward the OMCT is the quality of life (QOL) and associated adverse effects. It has been noted that the QOL in patients receiving OMCT in terms of functional scale like pain, difficulty in swallowing, dry mouth, mouth opening, sticky saliva, social eating, and social contact is significantly better from the baseline values at an interval between 3 and 6 months [17]**.** Also, the grade 3 or higher adverse events were less commonly noted in OMCT arm (19%) as compared to chemotherapy arm (30%). This difference is statistically significant, thus suggesting that OMCT as a good alternative to standard regimen in resource constrained situations (in terms of PFS, OS and adverse events [5, 14, 15].

### OMCT in Adjuvant and Neoadjuvant Settings

Complete surgical resection with R0 margins has been proven to be the most appropriate treatment option in oral cancers, especially in advanced oral cavity cancers. But to study the role of chemotherapeutic agents in resectable oral cancers, multiple studies have paved the way to present the aforementioned conclusion. Landmark studies in this field include studies by Licitra et al. and Zhong et al. [18, 19], both studies gave almost similar results, i.e., there was no difference in the OS and disease specific survival.

The above studies clearly made a line regarding the use of chemotherapeutic agents in induction settings in resectable oral cancers. But this interest was revived due to delay in definitive surgical treatment delivery to the patients, especially in developing countries like India where oral cancer is a major health problem. The delay can range from few weeks to a month or more which can very well turn a resectable oral cancer to borderline resectable or unresectable cancer and metastatic stage.

Chemotherapy agents and regimen can theoretically offer a useful way/approach to prevent progression during this waiting period. Although there is no randomized study to test this approach until now, there is some evidence from a study by Pai et al. The study compared two arms, i.e., one with direct surgery followed by adjuvant therapy and other arm included patients who received metronomic chemotherapy preoperatively followed by surgery and adjuvant therapy with maintenance OMCT. Two-year disease-free survivals (DFS) in OMCT group and control group was 86.5 and 71.6%, respectively. Also, the patients who received at least 3 months of adjuvant OMCT had 2 years DFS of 94.6% ^4^. Though short-term outcome in multiple studies by number of authors are similarly promising, we need to wait for long term results [20–23].

## Future Directions

This article attempts to highlight the current scenario of OMCT in relation to oral cavity squamous cell carcinoma. Majority of the studies present in the literature involves clinical outcomes, but further research is still required in terms of exact role of immunity in OMCT. Also, the biochemical interactions between the drugs in OMCT regimen and tumor microenvironment has not been elaborated and needs further investigations to ascertain the role of molecular pathways involved in the drug–tumor cell interactions.

It has been the established fact that chemotherapeutic agents, especially cisplatin sensitizes the tumor cells to effects of radiotherapy by mechanism of excessive oxidative overloading and sensitivity in G2 phase of cell cycle. Whether such mode of interaction exist in OMCT with radiotherapy is not clear and needs to be investigated on clinical as well as molecular backgrounds.

Currently few prospective trials are underway to answer the long-standing questions related to this drug regimen which includes: -

- Role of OMCT as adjuvant therapy post standard surgery and adjuvant therapy in locally advanced oral cancers (CTRI/2017/02/007777).
- Role of OMCT as adjuvant therapy in pharyngeal and laryngeal cancers post chemoradiation therapy (CTRI/2016/09/007315).
- Role of OMCT as neoadjuvant and maintenance therapy alongside standard surgery and adjuvant therapy in operable stage III/IV oral cancers (CTRI/2015/01/005405).
- Comparative study between OMCT and intravenous chemotherapy for patients with metastatic, recurrent, inoperable head and neck cancers with palliative intent treatment (CTRI/2015/11/006388).

These trials once published will give a clearer picture of exact role of OMCT as neoadjuvant therapy, adjuvant maintenance therapy and as a palliative intent therapy in head and neck cancers.

## Conclusion

With current evidence, there is a definite role of OMCT in treatment of oral SCC. OMCT can be an alternative in patients who are not tolerable or affordable for standard palliative chemotherapy and also an option for patient who are waiting for surgery. However, results of ongoing and future studies on exact mechanism, indications and implications of this drug regimen would help in integration OMCT in current standard of therapy.

## References

[1] Sung H, Ferlay J, Siegel RL, Laversanne M, Soerjomataram I, Jemal A, Bray F (2021) Global cancer statistics 2020: GLOBOCAN estimates of incidence and mortality worldwide for 36 cancers in 185 countries. CA Cancer J Clin 71(3):209–249. 10.3322/caac.21660. (**Epub 2021 Feb 4 PMID: 33538338**) 33538338 10.3322/caac.21660

[2] Mummudi N, Agarwal J, Chatterjee S, Mallick I, Ghosh-Laskar S (2019) Oral cavity cancer in the Indian subcontinent—challenges and opportunities. Clin Oncol 31(8):520–528 10.1016/j.clon.2019.05.013 31174947

[3] Coelho K (2012) Challenges of the oral cancer burden in India. J Cancer Epidemiol 2012:1–17 10.1155/2012/701932 PMC3471448 23093961

[4] Banavali S, Vaidya A, Prabhash K, Pai P (2013) Oral metronomic scheduling of anticancer therapy-based treatment compared to existing standard of care in locally advanced oral squamous cell cancers: a matched-pair analysis. Indian J Cancer 50(2):135 23979205 10.4103/0019-509X.117024

[5] Patil V, Noronha V, Joshi A, Muddu V, Dhumal S, Bhosale B et al (2015) A prospective randomized phase II study comparing metronomic chemotherapy with chemotherapy (single agent cisplatin), in patients with metastatic, relapsed or inoperable squamous cell carcinoma of head and neck. Oral Oncol 51(3):279–286 25578869 10.1016/j.oraloncology.2014.12.002

[6] Prabhash K, Noronha V, Krishna M, Patil V, Joshi A, Banavali S (2013) Metronomic therapy: chemotherapy revisited. Indian J Cancer 50(2):142 23979206 10.4103/0019-509X.117027

[7] Simsek C, Esin E, Yalcin S (2019) Metronomic chemotherapy: a systematic review of the literature and clinical experience. J Oncol 2019:1–31 10.1155/2019/5483791 PMC6446118 31015835

[8] Ghiringhelli F, Menard C, Puig PE et al (2007) Metronomic cyclophosphamide regimen selectively depletes CD4+CD25+ regulatory T cells and restores T and NK effector functions in end stage cancer patients. Cancer Immunol Immunother 56(5):641–648 16960692 10.1007/s00262-006-0225-8 PMC11030569

[9] Parikh PM, Hingmire SS, Deshmukh CD (2016) Selected current data on metronomic therapy (and its promise) from India. S Asian J Cancer 05(02):037–047 10.4103/2278-330X.181623 PMC4873693 27275444

[10] Pandey A, Desai A, Ostwal V, Patil V, Kulkarni A, Kulkarni R et al (2016) Outcome of operable oral cavity cancer and impact of maintenance metronomic chemotherapy: a retrospective study from rural India. S Asian J Cancer 05(02):052–055 10.4103/2278-330X.181625 PMC4873695 27275446

[11] Ko SH, Choi GJ, Lee JH, Han YA, Lim SJ, Kim SH (2008) Differential effects of selective cyclooxygenase-2 inhibitors in inhibiting proliferation and induction of apoptosis in oral squamous cell carcinoma. Oncol Rep 19:425–433 18202791

[12] Vermorken JB, Remenar E, van Herpen C, Gorlia T, Mesia R, Degardin M et al (2007) Cisplatin, fluorouracil, and docetaxel in unresectable head and neck cancer. N Engl J Med 357:1695–1704 17960012 10.1056/NEJMoa071028

[13] Klochikhin A, Greil R, Cohen E, Vermorken J, Harrington K, Tahara M et al (2015) Phase 3 trial of pembrolizumab as a first-line treatment in subjects with recurrent/metastatic head and neck squamous cell carcinoma: KEYNOTE-048. Ann Oncol 26:viii5 10.1093/annonc/mdv514.01

[14] Patil V, Noronha V, Dhumal S, Joshi A, Menon N, Bhattacharjee A et al (2020) Low-cost oral metronomic chemotherapy versus intravenous cisplatin in patients with recurrent, metastatic, inoperable head and neck carcinoma: an open-label, parallel-group, non-inferiority, randomised, phase 3 trial. Lancet Glob Health 8(9):e1213–e1222 32827483 10.1016/S2214-109X(20)30275-8

[15] Patil V, Noronha V, Joshi A, Pinninti R, Dhumal S, Bhattacharjee A et al (2015) Metronomic chemotherapy in platinum-insensitive failures and/or early failures postmultimodality management in oral cancers. Indian J Med Paediatr Oncol 36(03):161–165 26855524 10.4103/0971-5851.166725 PMC4743183

[16] Vermorken JB, Mesia R, Rivera F, Remenar E, Kawecki A, Rottey S et al (2008) Platinum-based chemotherapy plus cetuximab in head and neck cancer. N Engl J Med 359:1116–1127 18784101 10.1056/NEJMoa0802656

[17] Kandipalli S (2018) Impact of metronomic chemotherapy on quality of life in recurrent, residual and metastatic head & neck cancers. J Med Sci Clin Res 6(12):1 10.18535/jmscr/v6i12.11

[18] Licitra L, Grandi C, Guzzo M et al (2003) Primary chemotherapy in resectable oral cavity squamous cell cancer: a randomized controlled trial. J Clin Oncol 21:327–333 12525526 10.1200/JCO.2003.06.146

[19] Zhong L-P, Zhang C-P, Ren G-X et al (2013) Randomized phase III trial of induction chemotherapy with docetaxel, cisplatin, and fluorouracil followed by surgery versus up-front surgery in locally advanced resectable oral squamous cell carcinoma. J Clin Oncol 31:744–751 23129742 10.1200/JCO.2012.43.8820 PMC5569675

[20] Chiang S, Velmurugan B, Chung C, Lin S, Wang Z, Hua C et al (2017) Preventive effect of celecoxib use against cancer progression and occurrence of oral squamous cell carcinoma. Sci Rep 7(1):6235 28740192 10.1038/s41598-017-06673-3 PMC5524966

[21] Prabhash K, Patil V, Noronha V, Joshi A, Nayak L, Pande N et al (2017) Retrospective analysis of palliative metronomic chemotherapy in head and neck cancer. Indian J Cancer 54(1):25 29199656 10.4103/ijc.IJC_161_17

[22] Patil V, Prabhash K, Banavali S, Noronha V (2012) Metronomic chemotherapy in advanced oral cancers. J Cancer Res Ther 2(8):S106–S110 10.4103/0973-1482.92223 22322727

[23] Senthil Kumar K, Armugam V (2019) outcome of oral metronomic therapy with methotrexate and celecoxib in advanced/recurrent head and neck squamous cell carcinoma. Int J Sci Stud 6(12):203–208

[24] Prabhash K, Patil V, Noronha V, Joshi A, Agarwala V, Muddu V et al (2017) Comparison of paclitaxel-cetuximab chemotherapy versus metronomic chemotherapy consisting of methotrexate and celecoxib as palliative chemotherapy in head and neck cancers. Indian J Cancer 54(1):20 29199655 10.4103/ijc.IJC_160_17

[25] Hsieh M, Chen G, Chang D, Chien S, Chen M (2018) the impact of metronomic adjuvant chemotherapy in patients with advanced oral cancer. Ann Surg Oncol 25(7):2091–2097 29721725 10.1245/s10434-018-6497-3

